# Biogenic Synthesis of Ag Nanoparticles of 18.27 nm by *Zanthozylum armatum* and Determination of Biological Potentials

**DOI:** 10.3390/molecules27041166

**Published:** 2022-02-09

**Authors:** Muhammad Riaz, Muhammad Altaf, Pervaiz Ahmad, Mayeen Uddin Khandaker, Hamid Osman, Emad M. Eed, Yasmeen Shakir

**Affiliations:** 1 Sulaiman Bin Abdullah Aba Al-Khail—Centre for Interdisciplinary, Research in Basic Science (SA-CIRBS), Faculty of Basic & Applied Sciences, International Islamic University, Islamabad 44000, Pakistan; 2 Department of Chemistry, University of AJK, Muzaffarabad 13100, Pakistan; maltafs7860@yahoo.com; 3 Department of Physics, University of Azad Jammu and Kashmir, Muzaffarabad 13100, Pakistan; Pervaiz_pas@yahoo.com; 4 Centre for Applied Physics and Radiation Technologies, School of Engineering and Technology, Sunway University, Bandar Sunway 47500, Malaysia; mayeenk@sunway.edu.my; 5 Department of Radiological Sciences, College of Applied Medical Sciences, Taif University, Taif 21944, Saudi Arabia; ha.osman@tu.edu.sa; 6 Department of Clinical Laboratories Sciences, College of Applied Medical Sciences, Taif University, Taif 21944, Saudi Arabia; e.eed@tu.edu.sa; 7 Department of Biochemistry, Hazara University, Mansehra 21120, Pakistan; yasmeenshakir@hotmail.com

**Keywords:** *Zanthozylum armatum*, AgNPs, green synthesis, antibacterial, antioxidant

## Abstract

Nanotechnology has become a dire need of the current era and the green synthesis of nanoparticles offers several advantages over other methods. Nanobiotechnology is an emerging field that contributes to many domains of human life, such as the formulation of nanoscale drug systems or nanomedicine for the diagnosis and treatment of diseases. Medicinal plants are the main sources of lead compounds, drug candidates and drugs. This work reports the green synthesis of Ag nanoparticles (AgNPs) using the aqueous bark extract of *Zanthozylum armatum,* which was confirmed by a UV absorption at 457 nm. XRD analysis revealed an average size of 18.27 nm and SEM showed the particles’ spherical shape, with few irregularly shaped particles due to the aggregation of the AgNPs. FT-IR revealed the critical functional groups of phytochemicals which acted as reducing and stabilizing agents. The bark extract showed rich flavonoids (333 mg RE/g) and phenolic contents (82 mg GAE/g), which were plausibly responsible for its high antioxidant potency (IC_50_ = 14.61 µg/mL). Extract-loaded AgNPs exhibited the highest but equal inhibition against *E. coli* and *P. aeruginosa* (Z.I. 11.0 mm), whereas methanolic bark extract inhibited to a lesser extent, but equally to both pathogens (Z.I. 6.0 mm). The aqueous bark extract inhibited *P. aeruginosa* (Z.I. 9.0 mm) and (Z.I. 6.0 mm) *E. coli.* These findings—especially the biosynthesis of spherical AgNPs of 18.27 nm—provide promise for further investigation and for the development of commercializable biomedical products.

## 1. Introduction

Nanotechnology uses the processes of the separation, consolidation and deformation of materials by one atom or one molecule [1]. Factually, all metabolic reactions for the generation of energy and the extraction of waste products occurring efficiently at the subcellular level operate on the nanoscale. The emergence of microbial drug resistance has posed serious and numerous threats to human health worldwide. During the past few decades, the discovery of new antibiotics has become a prime R&D need; thus, scientists are now focused on exploring new horizons for combating these drug-resisting bacteria with new and effective antibiotics [2,3]. Nanobiotechnology is contributing to human life in various ways: for example, the diagnosis of diseases, drug delivery and nanomedicine. The advantageous applications of nanomaterials in biomedicine and industry are proven to be unmatched [4,5]. Nanoparticles possess entirely unique and advantageous properties because of their high surface area, which is directly proportional to their biological effectiveness [6]. Silver in colloidal form is of particular interest due to its good conductivity, chemical stability, and catalytic and antibacterial activities [7,8,9]. Before the discovery of antibiotics by Alexzander Fleming, silver had been the preferred choice of antimicrobial agent, and now AgNPs have received an enormous attention due to their extraordinary activities against a wide range of microorganisms including drug-resistant microbes, viruses and other eukaryotic microorganisms. In addition, at low concentrations, silver is mostly nontoxic to human cells [10,11,12,13,14]. These properties of silver make it a choice for various applications, including air and water treatment, and a wide range of material and biomedical applications, e.g., cancer treatments, antibiotic products, diabetic healing, dentistry, stem cells, electronics used in disease diagnosis, and food packaging [15,16,17,18,19,20]. Silver compounds (e.g., silver sulfadiazine cream) have also been used to treat burns and various infections [11,21,22]. Amenitop (silica gel microspheres containing a Ag–thiosulfate complex) is mixed into plastics for long lasting antibacterial protection, disinfecting filters and coating materials [23,24,25,26,27]. Chemical methods for the generation of nanoparticles carry a variety of disadvantages including cost and impacts on the environment and human health, which can be avoided by the green synthesis of these critically required nanomaterials [28,29].

*Zanthoxylum armatum* DC (common name: timber; family: Rutaceae) grows in the Himalayas at a height of 1000–2100 m in Pakistan, India and Nepal [30]. Various parts of *Z. armatum* are carminative and treat stomachache, toothache, dyspepsia, asthma, varicose veins, indigestion, diarrhea, bronchitis, cholera and helminthiasis, as well as various infections and inflammations [30,31]. In addition, its different parts are used as condiments, flavoring agents and aromatic tonic for fever [30]. The *Z. armatum* is a rich reservoir of flavonoids, lignans, coumarins, alkaloids and other conjugated phenolic natural products which are responsible for the synthesis of Ag nanomaterials, their stabilization and reduced toxicity. Although silver has been used and has a higher tolerance in biological systems, it still binds with unwanted proteins, fatty acids and DNA, resulting in side effects. It has been validated that the phytochemicals are capable of chelating with Ag nanomaterials, and possess higher radical scavenging properties, therefore require more attention because their chelation not only stabilizes these nanoparticles but also reduces their off-target binding and captures the produced reactive oxygen species (ROS). In addition, it has been validated that the smaller size and spherical shape of AgNPs lead to the much stronger antimicrobial and antiviral properties that are required to develop potential anti-infectious biomedical products. Therefore, we designed and undertook an exploration of the green synthesis of AgNPs, which should be of smaller size, stabilized and of spherical shape, to contribute to the potential development of marketable anti-infectious biomedical products.

## 2. Materials and Methods

### 2.1. Chemicals and Plant Material

*Z. armatum* (plant material) was collected from Muzaffarabad, AJK, Pakistan. The sample was identified in the Dept. of Botany U-AJK, Muzaffarabad, Pakistan, where the specimen (MR-MA-05-BOT-2017) is kept. The powder of its fresh and dried bark was utilized for the preparation of both extracts. Methanol, acetone, DMSO (dimethyl sulfoxide), aluminum chloride (AlCl_3_), sodium nitrite (NaNO_2_)_,_ DPPH (2,2-diphenyl-1-picrylhydrazyl), sodium carbonate (Na_2_CO_3_), ascorbic acid, gallic acid, rutin hydrate, Folin–Ciocalteu Reagent, and nutrient agar were purchased from Sigma-Aldrich, Burlington, MA, USA.

### 2.2. Preparation of Extracts

For the aqueous extract, the dried bark of *Z. armatum* was converted into a fine powder, of which 50 g was added to 300 mL of deionized water and kept at RT for 15 days. The resulting extract after filtering by Whatman No.1 filter paper was used for preparing the AgNPs. For the methanolic bark extract, 50 g of its powder was added to 300 mL of methanol and kept for 15 days at RT. The obtained extract, after filtering (Whatman No.1 filter paper) and drying by rotary evaporator under reduced pressure, was used for the experiments.

### 2.3. Synthesis of AgNPs

Green preparation of the AgNPs was achieved by using 100 mL of the AgNO_3_ solution and 100 mL of the aqueous bark extract of the Z. *armatum* (from 300 mL of the prepared extract). A color change from brownish to dirty brown revealed the green synthesis of the desired AgNPs, and this reaction mixture was left for 24 h at RT. A UV-Vis spectrophotometer was employed to verify the formation of the desired AgNPs, which were then purified through successive centrifugation at 3000 rpm for 15 min, dried, characterized and studied.

### 2.4. Characterization of AgNPs by UV-Vis, XRD, SEM and FT-IR

After observing the color change of the solution, spectra of the Analytik Jena SPECORD 50 UV-Visible Spectrometer were used for further confirmation of NPs, FT-IR (Perkin Elmer Spectrum 100) (Waltham, MA, USA) for functional group identification, X-ray diffraction analysis (Bruker D8 powder X-ray diffractometer, operating at a voltage of 40 KV and a current of 30 mA with K*α* Cu radiation) for the phase identification and characterization of crystalline nature/properties, and high-resolution SEM (JEOL JSM-6490A) were used for the morphological characterization of the size distributions of the AgNPs. Their particle sizes were determined by the Debye–Scherrer equation: D = 0.89 λ/*β*cosθ, where D is the average size of the synthesized AgNPs, K is the constant (K = 0.89), λ is the X-ray wavelength (0.1546 nm), *β* is the width of the maximum peak at ½ of the height and θ is the diffraction angle (in degrees).

### 2.5. Antibacterial Activities

The AgNPs, aqueous and methanolic extracts of *Z. armatum* were studied for antimicrobial activities using well diffusion method against *P. aeruginosa* and *E. coli* [32]. Cultures were subcultured on Muller-Hinton broth at 35 °C at 200 rpm. Every strain was uniformly swabbed on the respective plates under sterile conditions. Wells of 6 mm were made and 30 µL of each sample was added into the respective wells. Dimethyl sulfoxide (DMSO) was used as a negative control. Incubation was done at 35 °C for 24 h and zones of inhibition were measured in millimeters.

### 2.6. Antioxidant Activity

The methanolic bark extract of *Z. armatum* was subjected to antioxidant activity using DPPH-assay [33]. An amount of 7.98 mg of DPPH dissolved in 100 mL of methanol was used as a stock solution and three concentrations (10, 20, 30 µg/µL) of methanolic extract (1 mg/mL) were mixed with the DPPH solutions. The volume was made up with methanol. After incubation in the dark for 1 h, the absorbance was measured at 517 nm using the UV-Vis spectrophotometer, with ascorbic acid as a positive control. Measurements were done in triplicate. Percentage of radical scavenging activity was calculated by:

Inhibition (%) = (A_control_ − A_test_)/A_control_ × 100

where A_control_ = absorbance of ascorbic acid and A_test_ = absorbance of the test sample.

To find the IC_50_, different concentrations of the extract were plotted against their percentage of scavenging activity using standard linear regression in Microsoft Excel. A low IC_50_ value of the sample demonstrates that it has high antioxidant potential and vice versa.

### 2.7. Total Phenolic Contents

Total phenolic contents (TPCs) of the methanolic bark extract were determined by the Folin–Ciocalteu method [34]. Briefly, 200 µL of methanolic crude extract (1 mg/mL) of *Z. armatum* was made up to 3 mL with distilled water, mixed thoroughly with 0.5 mL Folin–Ciocalteu reagent and incubated for 3 min, followed by the addition of 2 mL of 20% sodium carbonate. This mixture was allowed to stand for a further 60 min in the dark and absorbance was measured at 650 nm. TPCs of the *Z. armatum* bark extract were calculated from the calibration curve (y = 1.56x − 0.075, R^2^ = 0.998) of the gallic acid and the results were expressed as milligrams of gallic acid equivalent per g dry weight (mg GAE/g dry weight).

### 2.8. Total Flavonoid Contents (TFCs)

TFCs of the methanolic bark extract of *Z. armatum* were determined by the AlCl_3_ colorimetric method [35]. In brief, 50 µL of crude extract (1 mg/mL of methanol) was mixed with 4 mL of distilled water, and then 0.3 mL of 5% NaNO_2_ solution and 0.3 mL of 10% AlCl_3_ solution were added after 5 min of incubation. This mixture was then allowed to stand for 6 min. Then, 2 mL of NaOH (1 M) solution was added and the final volume of the mixture was brought to 10 mL with distilled water. This mixture was allowed to stand for 15 min and the absorbance was measured at 510 nm. TFCs were calculated from a calibration curve (y = 0.404x − 0.026, R^2^ = 0.997) and the results were expressed as mg rutin equivalent per gram of dry weight of the samples (mg RE/g). Rutin was used as standard and the experiments were carried out in triplicate.

## 3. Results and Discussion

### 3.1. Visual Observation

**Figure 1:**
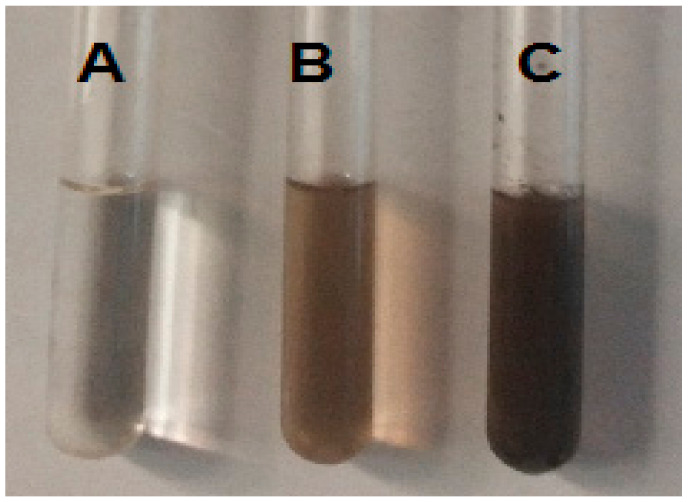
(**A**) AgNO_3_ aq. solution; (**B**) Aq. roots extract of *Z. armatum*; (**C**) AgNPs formed.

After 30 min, the brownish color of extract changed into dirty brown upon mixing the aqueous AgNO_3_ solution with the aqueous bark extract of *Z. armatum* (Figure 1). This color change was the first indication of AgNP formation by the aqueous bark extract due to surface plasmon resonance (SPR).

### 3.2. UV-Visible Spectroscopy

**Figure 2:**
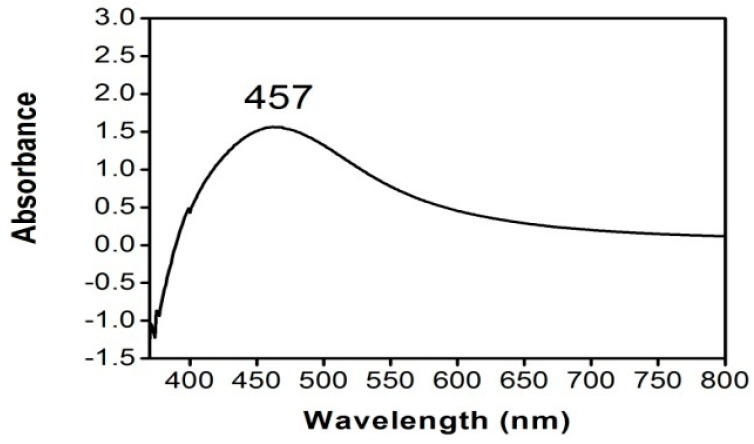
UV-Vis spectrum of biosynthesized AgNPs.

The UV-Vis spectroscopy was the preliminary technique for the characterization of the AgNPs. The reduction of pure Ag^+^ ions was monitored by measuring the UV-Vis spectrum of the reaction medium after 24 h by diluting a small aliquot of the sample in distilled water. Reduction of the Ag^+^ ions in the aqueous solution of the nanoparticles was confirmed by UV-Vis spectrum of the colloidal solution which exhibited a strong absorption at 457 nm due to the presence of surface plasmon resonance of the AgNPs (Figure 2). 

### 3.3. XRD Analysis

**Figure 3:**
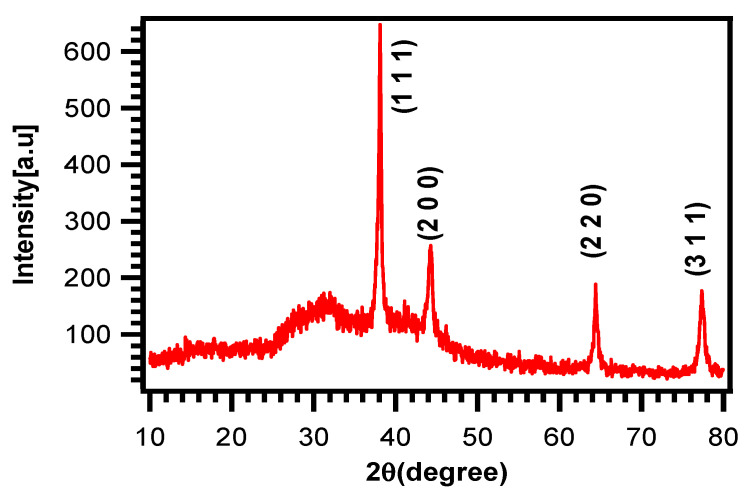
XRD pattern of biosynthesized AgNPs.

The XRD pattern of these AgNPs (Figure 3) was used to assess their size, phase identification and crystalline nature. Four intense and sharp peaks at 2θ = 38.096, 44.257, 64.390° and 77.311° can be indexed to the (1 1 1), (2 0 0), (2 2 0) and (3 1 1) planes of Bragg’s reflection of silver, respectively. The XRD pattern revealed the crystalline nature with the face-centered cubic structure of these Ag nanoparticles and the average crystallite size was found (18.27 nm), calculated using the Debye–Scherrer formula corresponding to the (1 1 1) plane with the cubic structure.

$$D=\frac{K\lambda }{\beta cos\theta }$$

where D = average particle size, *K* = particle shape factor (0.89), *λ* = wavelength of the X-ray beam used (0.154 nm), *β* = full width at half maximum (FWHM) of the (1 1 1) plane and *θ* = Bragg’s angle. For the peak corresponding to the (1 1 1) plane, the lattice constant ‘*α*’ is  4.086 A. These data are in agreement with the literature [25]. 

### 3.4. SEM Analysis

**Figure 4:**
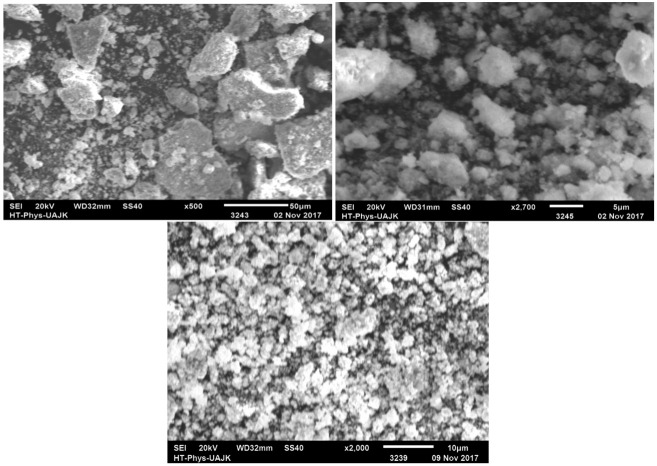
SEM micrographs of biosynthesized AgNPs.

The morphological characteristics were confirmed by scanning electron microscopy (SEM) (using a JEOL JSM-6490A Analytical Scanning Electron Microscope), Tokyo, Japan. The SEM images (Figure 4) revealed the high intensity of these spherical nanoparticles; some were irregularly shaped, without any defined morphology, while a few were dispersed and some others were aggregated. These results match with previous reports [36].

### 3.5. FT-IR Analysis

**Figure 5:**
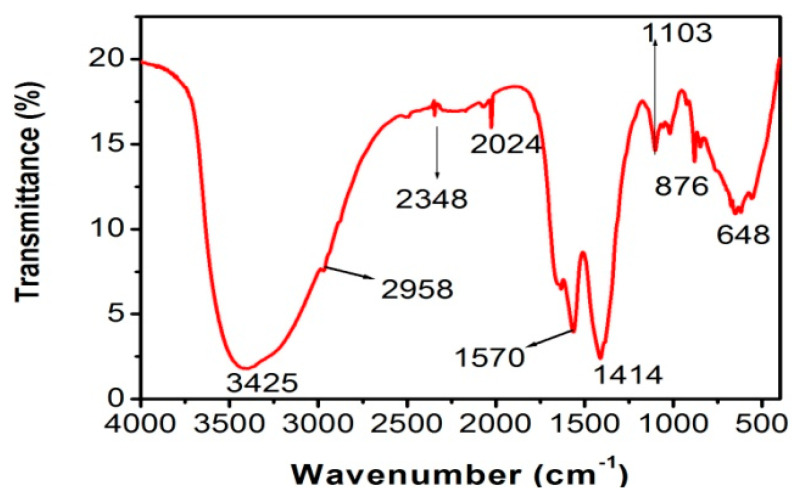
FT-IR spectrum of biosynthesized AgNPs.

FT-IR analysis was carried out to identify the functionalities of the phytochemicals in the *Z. armatum* extract (Figure 5). The phytochemicals in the bark extract displayed peaks at 3425, 2958, 2348, 2024, 1640 1570, 1414, 1103, 1019, 876 and 648 cm^−1^. Among all these peaks, the broad band at 3425 cm^−1^ was correlated with the OH-group of phenols and alcohols and overlapped with the N-H stretch of the amines, whereas the small bands at 2958 and 1414 cm^−1^ were due to the C-H symmetrical stretching and bending vibrations of the saturated hydrocarbons, respectively. The bands at 2024 and 1103 cm^−1^ were due to the C-N bending and stretching of the amides, respectively. The weak absorption band at 1640 cm^−1^ indicated the C=O stretch of the amide (which was not labeled in the spectra). The band at 1570 cm^−1^ was due to the N-H bend of the amines. The small band at 1019 cm^−1^ represented the C-O bond of the alcohols and phenols. The bands at 876 cm^−1^ indicated the presence of an aromatic C=C group. The strong peak at 648 cm^−1^ indicated the C-X groups of the alkyl halides. The O–H stretching vibration in the phenol and alcohol was broadened due to an overlap with the N-H and the presence of extensive H-bonding. The phenols and flavonoids present in the bark extract are powerful reducing and capping agents which may have been responsible for the green synthesis of these AgNPs by redacting AgNO_3_ [37].

### 3.6. Antibacterial Activity

**Figure 6:**
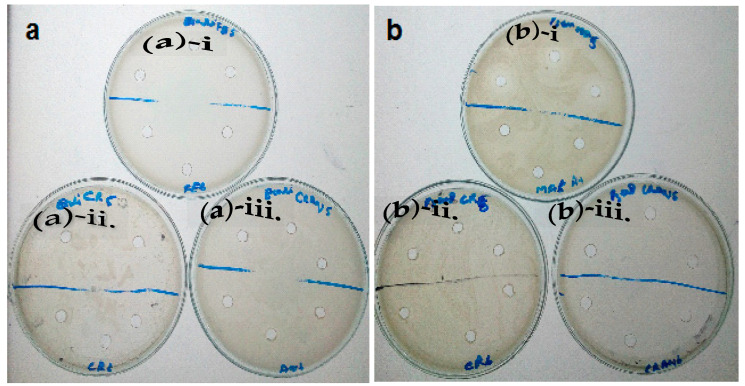
(**a**)**-i**. Antibacterial activity of AgNPs with *E. coli*; (**a**)**-ii.** Antibacterial activity of aq. extract with *E. coli*; (**a**)**-iii.** Antibacterial activity of MeOH extract with *E. coli*; (**b**)**-i.** Antibacterial activity of AgNPs with *P. aeruginosa*; (**b**)**-ii.** Antibacterial activity of aq. extract with *P. aeruginosa*; (**b**)**-iii.** Antibacterial activity of MeOH extract with *P. aeruginosa*.

An antibacterial screening of the bark extracts of *Z. armatum* and biogenically synthesized AgNPs was carried out for *Escherichia coli* (*E. coli*) and *Pseudomonas aeruginosa* (*P. aeruginosa*). Extract-loaded AgNPs showed the highest but equal inhibition against both *E. coli* and *P. aeruginosa* (Z.I. 11.0 mm), while the methanolic bark extract inhibited to a lesser extent but equally to both pathogens (Z.I. 6.0 mm). The aqueous bark extract inhibited *P. aeruginosa* (Z.I. 9.0 mm) and *E. coli* (Z.I. 6.0 mm) (Figure 6a,b).

### 3.7. Antioxidant Activity

**Figure 7:**
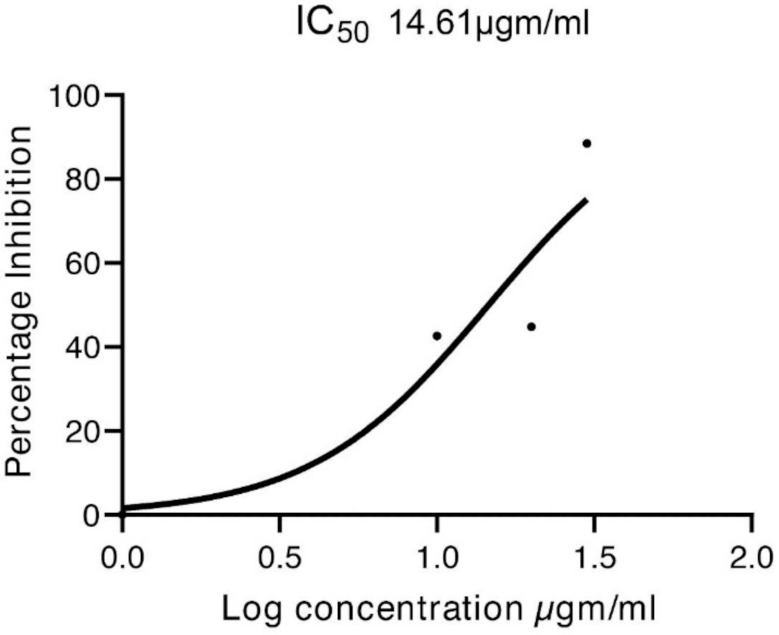
Calibration curve.

Plants rich in secondary metabolites, such as phenols and flavonoids, showed high antioxidant activities due to their redox properties. The antioxidant activity of *Z. armatum* was calculated in terms of IC_50_ values. The methanolic bark extract of *Z. armatum* showed strong antioxidant activity (IC_50_ = 14.61 µg/mL), indicating high free radical scavenging activity (Table 1 and Figure 7). Furthermore, the extracts proved to contain a high number of phenolic and flavonoid compounds, which were found to have a strong positive correlation with the free radicals’ (DPPH and OH) scavenging efficacies [38]. 

### 3.8. Phenolic and Flavonoid Contents

The total phenolic contents (TPCs) of the methanolic bark extract of *Z. armatum* (82 mg gallic acid equivalents/g were calculated from the calibration curve (R^2^ = 0.998)), and TFCs 333 mg rutin equivalents/g were also calculated from the calibration curve (R^2^ = 0.997) (Table 2). Flavonoids suppressed reactive oxygen formation, chelated the trace elements involved in free radical production, scavenged reactive species, upregulated and protected antioxidant defenses [39]. The high antioxidant potential is also due to the presence of flavonoids and phenolic moieties which inhibit free radicals. The results are shown as the mean values of the three replicates.

## 4. Conclusions

The bioreduction of Ag ^+^ into Ag^0^ (AgNPs) in aqueous solutions of silver nitrate by the natural products of *Z. armatum* extract was indicated by UV-Vis absorptions. The XRD analysis showed the FCC structure of these Ag nanoparticles and provides the particle size of 18.27 nm. SEM showed their spherical shape, in addition to some of their aggregates. The FT-IR spectra confirmed the bioreduction of Ag ^+^ ions into Ag^0^, plausibly by the phenols and flavonoids present in the aqueous extract of *Z. armatum*. These nucleophilic aromatic moieties, phenols and flavonoids are responsible for the chelation that stabilizes these AgNPs. The methanolic extract of *Z. armatum* showed high phenolic (82 mg GAE/g) and flavonoid (333 mg RE/g) content, which was presumably responsible for the attractive antioxidant efficacy (IC_50_ = 14.61 µg/mL). This study concludes that these AgNPs carry the highest antimicrobial activity, while the aqueous extract showed intermediate antimicrobial activity, and the methanolic extract did not exhibit any antimicrobial activity against both pathogens. This research provides a substantial contribution to the development of antioxidant and anti-infectious biomedical products because: (1) it demonstrated the synthesis of small-sized AgNPs, which is required for higher efficacy; (2) it demonstrated the spherical shape of synthesized AgNPs, and spherical AgNPs are more active against microbes and viruses; (3) synthesized AgNPs were found to be stabilized due to the chelation of phytochemicals found in *Z. armatum*, plausibly due to the flavonoids and phenolic natural products; and (4) these chelating phytochemicals are expected to reduce the off-target bindings of these therapeutic AgNPs.

## Figures and Tables

**Table 1:** Antioxidant activity of the methanolic bark extracts of *Z. armatum*.

**Conc. of MeOH-Extracts**	10 µg/µL	20 µg/µL	30 µg/µL
**% Scavenging**	42.58	44.84	46.65

**Table 2:** Total phenolic and flavonoid contents of methanolic bark extract of *Z. armatum*.

#	Total Phenolic and Flavonoid Contents	mg (GAE or RE)/g DW
1	Total phenolic content	82
2	Total flavonoid content	333

## Sample Availability

Samples of the compounds are available from the authors.
